# Large-Scale Genomic Analyses and Toxinotyping of *Clostridium perfringens* Implicated in Foodborne Outbreaks in France

**DOI:** 10.3389/fmicb.2019.00777

**Published:** 2019-04-17

**Authors:** Abakabir Mahamat Abdelrahim, Nicolas Radomski, Sabine Delannoy, Sofia Djellal, Marylène Le Négrate, Katia Hadjab, Patrick Fach, Jacques-Antoine Hennekinne, Michel-Yves Mistou, Olivier Firmesse

**Affiliations:** Université PARIS-EST, Agence Nationale de Sécurité Sanitaire de l’Alimentation, de l’Environnement et du Travail (ANSES), Laboratory for Food Safety, Maisons-Alfort, France

**Keywords:** foodborne outbreak, *Clostridium perfringens* enterotoxin, virulence gene profiles, virulence factors, real-time PCR, whole genome sequencing, coregenome SNP

## Abstract

*Clostridium perfringens* is both an ubiquitous environmental bacterium and the fourth most common causative agent of foodborne outbreaks (FBOs) in France and Europe. These outbreaks are known to be caused by *C. perfringens* enterotoxin (CPE) encoded by the *cpe* gene. However, additional information on the toxin/virulence gene content of *C. perfringens* has become available in the last few years. Therefore, to understand the enteropathogenicity of this bacterium, we need to describe the toxin and virulence genes content of strains involved in FBOs. In this study, we used a new real-time PCR typing technique based on a comprehensive set of 17 genes encoding virulence factors. The analysis was performed on a collection of 141 strains involved in 42 FBOs in the Paris region. It was combined with whole genome sequence (WGS) phylogenomic reconstruction, based on the coregenome single nucleotide polymorphisms (SNPs) of 58 isolates, representatives of the identified virulence gene profiles. Two or three different virulence gene profiles were detected in 10 FBOs, demonstrating that *C. perfringens* FBOs may be associated with heterogeneous strains. *cpe-*positive strains were isolated in 23 outbreaks, confirming the prominent role of CPE in pathogenicity. However, while *C. perfringens* was the sole pathogen isolated from the incriminated food, the *cpe* gene was not detected in strains related to 13 outbreaks. This result indicates either that the standard method was not able to isolate *cpe+* strains or that the *cpe* gene may not be the only determinant of the enterotoxigenic potential of *C. perfringens* strains. Using phylogenomic reconstruction, we identified two clades distinguishing chromosomal *cpe*-positive from *cpe*-negative and plasmid-borne *cpe.* Important epidemiological information was also garnered from this phylogenomic reconstruction that revealed unexpected links between different outbreaks associated with closely related strains (seven SNP differences) and having common virulence gene profiles. This study provides new insight into the characterization of foodborne *C. perfringens* and highlights the potential of WGS for the investigation of FBOs.

## Introduction

*Clostridium perfringens* is a Gram-positive, spore-forming, anaerobic, rod-shaped bacterium, known as an important causative agent of foodborne and non-foodborne gastroenteritis (Grass et al., 2013). The ability of this bacterium to form resistant spores contributes to its survival in many environmental niches, including soil, sewage, foods, and the intestinal microbiota of humans and animals (Xiao, 2014; Li et al., 2016). *C. perfringens* can cause necrotic enteritis, enterotoxemia, and gastroenteritis in animals (Keyburn et al., 2008; Uzal et al., 2015). It is also a foodborne pathogen that causes diarrhea and abdominal pain in humans (Scallan et al., 2011). *C. perfringens* is commonly found as a contaminant in meat and poultry products, as well as in vegetables and crops. This bacterium is an important pathogen involved in foodborne outbreaks (FBOs) and ranks as the fourth most common causative agent of foodborne illness due to bacterial toxins in France and Europe, with more than 1,500 human cases each year (EFSA, 2016). The pathogenicity of this bacterium is largely attributable to its ability to produce a variety of virulence factors. These virulence factors include well-characterized pathogenic toxins and hydrolytic enzymes. Their roles in FBO remain to be defined but their presence is an aggravating factor (Li et al., 2016). Toxin production varies among *C. perfringens* strains and is the basis for the classification system, which is based on the production of four major toxins, namely CPA, CPB, ETX, and ITX that divide *C. perfringens* strains into toxinotypes A to E (McClane et al., 2013). This classification was recently revised to include two additional toxinotypes, i.e., *C. perfringens* toxinotype F strains producing enterotoxin (CPE) but not CPB, ETX or ITX, and *C. perfringens* toxinotype G strains producing NetB (Rood et al., 2018) (Table 1). The number of characterized virulence factors is constantly increasing, with more than 20 toxins and hydrolytic enzymes identified to date (Li et al., 2016; Kiu and Hall, 2018). However, there is considerable variability in the toxin armamentarium, and a single strain cannot produce all these virulence factors (Freedman et al., 2016). The virulence factors of *C. perfringens* can be classified functionally as membrane-damaging enzymes, pore-forming toxins, intracellular toxins, and hydrolytic enzymes (Revitt-Mills et al., 2015). Genes encoding these virulence factors may be located on the chromosome, and on the large plasmid (Freedman et al., 2016). Chromosomal virulence factors include the *cpa* gene (encoding α-toxin), *colA* (κ-toxin), *pfoA* (θ-toxin), and *nagH* (hyaluronidase or μ-toxin). These genes are located on the variable region of the chromosome (Chalmers et al., 2008a). The *nanH*, *nanI*, and *nanJ* genes encoding different sialidases, and *cadA* encoding ν-toxin are located on a conserved region of the chromosome (Obana and Nakamura, 2011). The genes *cpb* (encoding CPB-toxin), *cpb2* (CPB2-toxin), *etx* (ETX-toxin), *ia/ib* (ITX-toxin), *netB* (NetB-toxin), *tpeL* (TpeL-toxin), *ureABC* (ureases), *cpd* (δ-toxin), *lam* (λ-toxin), and *becA/becB* encoding the binary enterotoxin for *C. perfringens* are located on large plasmids of variable size ranging from 65 to 110 kb (Li et al., 2013). Recently, partial genome sequencing of the canine isolate revealed three novel putative toxin genes encoding proteins related to the pore-forming leucocidin/hemolysin family. These putative toxin genes were designated *netE*, *netF*, and *netG* (Mehdizadeh Gohari et al., 2015). *netE* and *netF* are located on a large conjugative plasmid, and *netG* is located on another large plasmid (Mehdizadeh Gohari et al., 2017). The *C. perfringens* enterotoxin gene (*cpe*) can be located on either the chromosome or on the plasmid (Gao and McClane, 2012). A strain harboring both a chromosomal and plasmid-borne *cpe* gene has not yet been observed (Freedman et al., 2016). *C. perfringens* enterotoxin (CPE) is one of the toxins associated with sporulation and has been implicated in FBOs and non-foodborne gastroenteritis illnesses (Lukinmaa et al., 2002).

**Table 1:** Revised classification of *C. perfringens* based on the production of six major toxins (Rood et al., 2018).

Type	Toxin produced					
	
	α(CPA)	β(CPB)	ε(ETX)	ι(ITX)	CPE	NetB
A	+	-	-	-	-	-
B	+	+	+	-	-	-
C	+	+	-	-	±	-
D	+	-	+	-	±	-
E	+	-	-	+	±	-
F	+	-	-	-	+	-
G	+	-	-	-	-	+


*+, toxin produced; -, toxin not produced.*

The Standardized method NF EN ISO 7937 is the conventional method for detecting *C. perfringens* strains in foods by the enumeration of bacterial colonies on specific agar media (Anonymous, 2005). With this robust method, it is possible to detect and enumerate viable *C. perfringens* in food samples and to obtain isolates that can be characterized further. This reference method therefore makes it possible to isolate the bacterium but does not provide any phenotypic information distinguishing the strains from one another, particularly with regards to their virulence (Law et al., 2015). While the detection of CPE in the stools of patients is a marker for implicating *C. perfringens* as the etiological agent in foodborne illnesses, CPE is produced in the intestine during sporulation of vegetative cells and is consequently absent from the contaminated food (Lin and Labbe, 2003). Moreover, isolation of *cpe-*positive *C. perfringens* in suspected foods and from stools of affected individuals may be accompanied by the presence of *cpe-*negative *C. perfringens* from the environment and microbiota (Scallan et al., 2011). This situation makes toxin detection hazardous to link isolates from patients to food vehicles (Lindström et al., 2011). The common assumption that the intestinal pathogenicity of *C. perfringens* relies on a specific toxin was recently challenged by findings suggesting that additional virulence factors may be involved in cytotoxicity (Yasugi et al., 2015). This prompted us to explore the virulence potential of *C. perfringens*, taking into account the more recently described virulence factors and phylogenomic inference presenting high discrimination power.

To improve epidemiologic knowledge about *C. perfringens* involved in FBOs, we developed a new real-time PCR typing technique targeting 17 genes encoding 15 virulence factors. The method was applied to 141 *C. perfringens* FBO strains isolated in the Paris region between 2013 and 2017. The virulence gene profiles obtained by this method would make it possible to describe strain diversity with regards to the *cpe* gene among these outbreaks, and robust phylogenomic analysis based on whole genome sequencing (WGS) data (Felten et al., 2017) would enable us to determine phylogenomic diversity at a large genomic scale, as recently emphasized (Kiu et al., 2017).

While many food pathogens have been sequenced and studied extensively, limited genome sequence data for *C. perfringens* are available in public databases, and very few genomic studies have been conducted on food isolates (Shimizu et al., 2002; Myers et al., 2006; Mehdizadeh Gohari et al., 2017; Lacey et al., 2018). To our knowledge, the largest genomic study conducted on 56 *C. perfringens* genomes contained only three foodborne strains (Kiu et al., 2017). Genomic comparison previously conducted on 12 strains from different toxinotypes, isolated from different sources, highlighted a lack of features differentiating toxinotype A from the other isolates, indicating that toxinotype A can easily shift to other types by acquisition of toxin-encoding plasmids (Hassan et al., 2015). A recent genomic study conducted on necrotic enteritis-causing isolates of *C. perfringens* demonstrated that pathogenicity does not correlate with coregenome content, but rather with accessory gene content located on the chromosome and plasmids (Lacey et al., 2018). To better understand the diversity among *C. perfringens* implicated in FBOs, we performed whole genome analysis on 58 *C. perfringens* strains selected on the basis of epidemiologic information and diversity of virulence gene profiles.

## Materials and Methods

### Microbiological and Epidemiological Data on Foodborne Outbreaks Associated With *C. perfringens* in the Paris Region

The microbiological and epidemiological data concerning each FBO (i.e., number of human cases, location, symptoms, and type of incriminated food) were collected by interviews or questionnaires managed by the local health authorities. The incriminated food was collected and sent to laboratories at ANSES (French Agency for Food, Environmental and Occupational Health and Safety) for microbiological analysis. We studied a collection of 141 isolates of *C. perfringens* involved in 42 FBOs from 2013 to 2017, including 1,267 human cases. Bacterial strains were isolated from suspected food by plating on selective Tryptose Sulfite Cycloserine (TSC) media according to the EN NF ISO 7937 standard method. The level of contamination by *C. perfringens* in the implicated foods was between 3.0 × 10^1^ and 1.0 × 10^6^ cfu/g. *C. perfringens* was isolated from 10 outbreaks in association with other bacterial species (including *B. cereus* and *S. aureus*) during microbiological investigations. Overall, vegetable dishes were the most common food reported (15 outbreaks, 36%), followed by poultry (10 outbreaks, 24%). Pork and beef sources were involved in practically the same proportion (six outbreaks, 14% for each) as other sources (five outbreaks, 12%) (Table 2).

**Table 2:** Microbiologic data for FBOs associated with *C. perfringens* from the Paris region between 2013 and 2017.

FBO	Year	Food vehicle	Human cases (*n*)	cfu/g	Pattern identified (*n*)	Virulence gene profile (pattern)	Recovered strain (*n*)	Other identified FBO bacteria	cfu/g
2207	2013	Vegetable	130	3.7E+06	1	XIII	1	-	-
2253	2013	Poultry	160	6.0E+06	1	XIII	1	-	-
435	2014	Other	22	3.0E+02	1	IV	1	-	-
1601	2014	Other	15	3.5E+05	1	IV	1	BC	2.7E+04
1622	2014	Other	21	1.1E+04	1	VIII	1	-	-
2370	2014	Pork	27	2.6E+03	1	V	1	-	-
529	2015	Vegetable	6	3.0E+01	2	X; IX	2	BC	1.5E+03
538	2015	Poultry	38	1.5E+04	1	V	7	-	-
2291	2015	Pork	118	4.0E+06	1	V	5	-	-
2318	2015	Poultry	34	1.1E+03	1	V	1	-	-
2540	2015	Vegetable	9	4.0E+02	2	VIII; X	2	BC	8.0E+03
2606	2015	Vegetable	9	3.7E+02	2	VIII; X	5	BC	1.0E+05
00000	2015	NK	5	NK	2	VIII; XIV	5	NK	NK
2727	2015	Pork	7	3.7E+03	2	IV; X	3	-	-
2773	2015	Poultry	50	1.5E+07	1	VII	5	-	-
2774	2015	Poultry	50	1.5E+07	1	VII	5	-	-
2987	2015	Poultry	39	3.6E+04	1	VII	8	-	-
2988	2015	Poultry	39	1.4E+02	2	VII; X	4	BC	1.4E+05
3803	2015	Pork	4	1.3E+04	2	X; XI	4	SCP	4.0E+02
3958	2015	Pork	NK	1.6E+02	1	XI	5	-	-
3863	2015	Vegetable	20	2.2E+02	3	VII; XIII; XV	5	-	-
4068	2015	Other	2	4.0E+04	1	X	2	-	-
4092	2015	Beef	30	1.2E+02	1	IV	4	-	-
4115	2015	Vegetable	2	3.6E+01	2	X; XI	2	-	-
4127	2015	Pork	26	5.8E+05	1	VI	4	-	-
4138	2016	Other	1	6.0E+02	1	X	3	-	-
370	2016	Vegetable	8	4.0E+01	1	III	1	BC	1.0E+03
490	2016	Poultry	40	7.0E+01	1	V	5	-	-
553	2016	NK	4	8.0E+02	1	XI	1	-	-
1781	2016	Vegetable	28	1.1E+05	1	XI	5	-	-
1782	2016	Vegetable	28	4.0E+01	1	XI	1	BC	5.5E+04
1923	2016	Vegetable	13	8.4E+04	1	VII	5	-	-
3199	2016	Vegetable	5	4.0E+01	1	X	1	-	-
3566	2016	Vegetable	51	2.4E+06	1	IV	5	-	-
4286	2016	Vegetable	2	4.0E+01	1	I	1	BC	4.0E+03
4430	2016	Beef	2	1.1E+03	1	VII	5	-	-
4493	2017	Vegetable	2	3.6E+02	3	X; XI; XII	4	BC	1.5E+04
4755	2017	Vegetable	120	9.3E+02	1	V	5	-	-
274	2017	Beef	2	4.0E+02	1	X	1	-	-
759	2017	Beef	31	4.9E+06	1	II	5	-	-
762	2017	Beef	31	7.0E+01	1	II	4	-	-
1270	2017	Beef	41	1.9E+03	1	X	5	-	-


*BC, *Bacillus cereus*; SCP, coagulase-positive *Staphylococcus aureus*; NK, not known.*

### DNA Extraction

With the aim of obtaining DNA for PCR and WGS-based applications, isolates of *C. perfringens* were revived from a frozen stored culture (-20°C) and anaerobically inoculated for 24 h at 37°C in Thioglycolate Broth with Resazurin^®^ (bioMérieux, Marcy l’Etoile, France). The subsequent culture was streaked on Columbia agar with 5% sheep blood (bioMérieux, Marcy l’Etoile, France) and incubated for 24 h at 37°C under anaerobic conditions using a Genbox anaer sachet^®^ (bioMérieux, Marcy l’Etoile, France).

Concerning PCR-based applications, DNA extraction was performed using the InstaGene kit (Bio-Rad Laboratories, Marnes-la-Coquette, France), following the manufacturer’s instructions for Gram-positive bacteria. For each culture, cells were washed by centrifugation at 17,000 × *g* for 3 min in 1 mL of phosphate buffer solution (PBS). The cell pellet was resuspended in 200 μL of Chelex-based solution and heated for 20 min at 56°C and then at 100°C for 10 min. The DNA solution was cooled for 5 min, and centrifuged at 17,000 × *g* for 3 min to eliminate cell debris. The purity of the extracted DNA solution was assessed using NanoDrop 2000 (Thermo Scientific, Villebon, France), according to the instructions provided by the manufacturer. The ratio of absorbance at 260 nm and 280 nm was used to assess protein contamination, while the ratio of absorbance at 260 nm and 230 nm was estimated to assess phenol contamination. The standard for purity was an A_260_/A_280_ ratio between 1.8 and 2.2, and an A_260_/A_230_ ratio between 2.0 and 2.2. The DNA was then diluted to obtain 3.3 × 10^2^ ng.μL^-1^, corresponding to 10^5^ genome equivalents. DNA solutions were stored at -20°C for further PCR analysis.

Concerning WGS-based applications, DNA was extracted using the Wizard Genomic DNA Purification kit (Promega, Charbonnières-les-Bains, France), according to the manufacturer’s instructions. Culture conditions and assessment of the quality and quantity of extracted DNA were performed as described above. Before library preparation, 200 ng of genomic DNA were analyzed on 0.8% agarose gel to ensure the absence of RNA contamination and DNA degradation.

### PCR Conditions

The BioMark^TM^ real-time PCR system (Fluidigm, San Francisco, CA, United States) was used for high-throughput microfluidic real-time PCR amplification using the 192×24 dynamic array (Fluidigm, San Francisco CA, United States). This chip dispenses 24 PCR mixes and 192 samples into individual wells, after which on-chip microfluidics assemble PCR reactions in individual chambers prior to thermal cycling resulting in 4,608 reactions.

Amplifications were performed using EvaGreen DNA binding dye (Biotium Inc., Hayward, CA, United States) with Perfecta qPCR tough mix (Quanta bio, Beverly, MA, United States), in accordance with the manufacturer’s recommendations. Three microliters of sample mix (containing 1.47 μL perfecta qPCR tough mix, 0.15 μL DNA binding dye sample loading reagent, 0.15 μL EvaGreen, 0.06 μL Rox reference dye, 0.76 μL DNA, and 0.41 μL DNase free water) were loaded into each sample inlet of the dynamic chip. Three μL of primer mix (containing 1.5 μL assay loading reagent, 0.75 μL primer stock containing 20 μM of each primer and 0.75 μL DNase free water) were loaded into each assay inlet of the dynamic chip. The thermal profile comprised 10 min at 95°C (Taq polymerase activation), followed by 35 cycles of 95°C for 15 s, and 60°C for 1 min (amplification steps), followed by melting curve analysis. The assays were performed in duplicate. Data were acquired on the BioMark^TM^ Real-Time PCR system and analyzed using Fluidigm Real-time PCR Analysis software to obtain crossing point (Ct) values. Two negative controls (no template controls) were included per chip and a pool of DNA for reference strains. CIP106156 known to be positive for the *cpa, cpe, ia, ib, pfoA, colA, lam, nagH, nanH, nanI, nanJ*, and *cadA* genes, CIP106527 positive for the *cpb1* and *tpeL* genes, and CIP104612 positive for the *cpb2* gene were used as positive controls. Targeted genes and corresponding primers are listed in Table 3.

**Table 3:** Primers used for the detection of 17 genes encoding 15 virulence factors.

Gene	Primer name	Sequence	References	Primer length	Amplicon (bp)
cpa	CPALPHTOX1TM-F	AAGAACTAGTAGCTTACATATCAACTAGTGGTG	Albini et al., 2008	33	124
	CPALPHTOX1TM-R	TTTCCTGGGTTGTCCATTTCC		21	
cpb	CPBEATOX-F	TGGAGCGTGAAAGAAACTGTTATTA	Albini et al., 2008	25	85
	CPBEATOX-R	GGTATCAAAAGCTAGCCTGGAATAGA		26	
cpb2	CPBEA2TOX-F	TATTTCAAAGTTTACTGTAATTTTTATGTTTTCA	Albini et al., 2008	34	127
	CPBEA2TOX-R	CCATTACCTTTCTATAAGCGTCGATT		26	
*etx*	CPETOXINTM-F	TTTGATAAGGTTACTATAAATCCACAAGGA	Albini et al., 2008	30	121
	CPETOXINTM-R	AGAGAGCTTTTCCAACATAAACATCTTC		28	
*iap*	CPIOTATM-F	GCATTAAAGCTCACACCTATTCCA	Albini et al., 2008	24	85
	CPIOTATM-R	GAGATGTGAGAGTTAATCCAAATTCTTG		28	
*ibp*	*ibp-F*	AGTTCCAAGTGACCAAGAAATAC	This study	23	182
	*ibp-R*	CCTGAATATTGCAAAGTTGCTTC		23	
*cpe*	*cpe-F*	ATAGATAAAGGAGATGGTTGGA	This study	22	178
	*cpe-R*	CCATATTCTACAGATGCTTGTA		22	
*pfoA*	*pfoA-F*	TGGAGCCTATGTTGCACAGT	This study	20	211
	*pfoA-R*	ATCTCTCCACCATTCCCAAG		20	
*netB*	*netB-F*	ACCGCTTCACATAAAGGTTGG	This study	21	160
	*netB-R*	TCAGGCCATTTCATTTTTCCGT		22	
*tpeL*	*tpeL-F*	GTGCCAATTGCAGGTATATCAAG	This study	23	247
	*tpeL-R*	ATCCTCCTTCCATTGCCCATA		21	
*colA*	*colA-L*	GGGCTTCAAAGGAAGTTAAGG	This study	21	220
	*colA-R*	TACTTTCCTCTGGTGTTCTTTCA		23	
*lam*	*lam-F*	CTGCAGTGAGCGCACATAGT	This study	20	231
	*lam-R*	CTCACTGCAGCTGGATCATTT		21	
*nagH*	*nagH-F*	TCATGGAGAATATATTGGGGTTA	This study	23	122
	*nagH-R*	TCCACTCAACACCATTCATAG		21	
*cadA*	*cadA-F*	AGATGCAGCCATAGAAGCTG	This study	20	198
	*cadA-R*	ACATCTCCAAATACAGCTTCC		21	
*nanH*	*nanH-L*	GGATAATGGTGAAACATGGAC	This study	21	117
	*nanH-R*	CCAGAGTAATCATATCTTGTAC		22	
*nanI*	*nanI-F*	AAGGTAAACAATCTAGTGCTGT	This study	22	82
	*nanI-R*	TCTATTATCATTTGGAGATTCTC		23	
*nanJ*	*nanJ-F*	TGTTTATAAAACACAACCAGTAG	This study	23	122
	*nanJ-R*	CATCTATAGAAGCTAAAACCGT		22	


### Strain Selection for WGS

Fifty-eight strains representative of all toxin gene profiles in all FBOs were selected for WGS. Additional isolates were selected to confirm the specificity and sensitivity of the PCR method to detect the 17 virulence genes. Three contaminated DNA samples of profile I (FBO 4286), profile X (FBO 2606), and profile XIV (FBO, NK) were removed from WGS analysis.

### Read Quality Check and Assembly

The genome sequencing was performed by the ‘Institut du Cerveau et de la Moelle épinière’ (ICM)^1^ (Hôpital de la Pitié-Salpêtrière, Paris) using NextEra XT libraries (Illumina, San Diego, CA, United States), indexed following the manufacturer’s recommendations (Illumina, San Diego, CA, United States), purified with the Agencourt AMPure XP system (Beckman Coulter), and controlled with Microfluidic Labchip GX (PerkinElmer, Villebon-sur-Yvette). These libraries were sequenced with a NextSeq 500 sequencer (Illumina, paired-end reads of 150 bases each). Assembly and variant calling of genomes were performed with an in-house workflow called ARtWork. Briefly, Paired-end reads stored as fastq.gz files were decompressed and BBMap (Bushnell, 2014) was used in order to estimate deep coverage. The reads with depth coverage lower than 20× were discarded from the workflow. Those higher than 100× were normalized at 100× using BBNorm (Xu et al., 2015). The quality of paired-end reads was evaluated with FASQC software v0.11.2. FASQC reports were produced for each sample of retained reads. Trimmomatic was used to trim reads less than 50 bp in length and bases presenting a phred score less than 20 (Bolger et al., 2014). The closest related reference genome of each sample was identified by estimating the Jaccard index (Ondov et al., 2016) against a collection of closest reference genomes (Supplementary Material) and was further used to perform scaffolding with MeDuSa (Bosi et al., 2015) and gap filling with GMcloser (Kosugi et al., 2015). The scaffolds of less than 200 bp in length were trimmed with Biopython (Cock et al., 2009). The quality of assemblies was assessed using QUAST metrics (Gurevich et al., 2013). Overall, the genomes showed the expected GC content of 28% and most of the contigs could be organized into single scaffolds. The sizes of the largest scaffolds ranged from 2.6 to 3.33 Mbp, with an average of 3.0 Mbp, in perfect agreement with the expected 3.0 Mbp size of the *C. perfringens* genome (Supplementary Material).

### Variant Calling Analysis

Also implemented in the ARtWork workflow, a variant calling analysis was performed using the iVARCall2 workflow (Felten et al., 2017), an in-house variant caller previously published and based on the HaplotypeCaller algorithm able to call single nucleotide polymorphisms (SNPs) and small insertions/deletions (InDels) at the coregenome scale simultaneously, and for each genome independently. The iVARCall2 workflow produces g.VCF files for each genome, which can be filtered and merged in order to produce high-quality variants and related pseudogenomes (i.e., VCFtoPseudogenome script). This pseudogenome corresponds to the reference genome where the genotypes of detected variants are replaced in each genome of the dataset. At the end of the iVARCall2 workflow, reports on breadth and depth coverage were produced, as well as matrices of pairwise distances. To assess quality of variant calling (Sandmann et al., 2017), mapping against the reference genome ATCC13124 was performed and resulted in breadth and depth coverages of 84 ± 7% and 389×, respectively.

### Coregenome Phylogenomic Inference

A maximum likelihood (ML) coregenome phylogenomic tree based on the pseudogenomes was constructed using the general time-reversible model with gamma distributed sites (GTRGAMMA), the secondary structure 16-state model, and a bootstrap analysis (*n* = 100) implemented in RaxML (Stamatakis, 2015). A total of 59,593 coregenome SNPs within 902 conserved core genes in all 58 genomes were identified in the dataset. Interactive Tree of Life (iTOL) was used to visualize the tree and annotate metadata (Letunic and Bork, 2016). In order to estimate the number of replicates necessary to yield accurate confidence values, a posterior bootstrap convergence test was computed with RAxML (Pattengale et al., 2010).

### *In silico* Detection of Virulence Factors

The coding sequences (CDSs) of 24 genes (including *becA, becB, ureABC*, and *cpd*) encoding virulence factors were retrieved from NCBI and queried against the 58 assembled genomes using nucleotide BLAST (identity = 90%; minimum coverage = 85%) to investigate the presence of these genes and confirm the results obtained by real-time PCR amplification.

### Statistical Analyses

Statistical analyses were conducted in R studio (version 2017-03-06). A Shapiro–Wilk test was performed to assess the normality of data, and a non-parametric Kolmogorov–Smirnov test was used to compare the diversity between phylogroups (Veshnyakova et al., 2012). The associations between different genes were tested using a Spearman’s rank correlation test (Myers and Sirois, 2004). The difference between the number of genes detected in *cpe-*positive strains and *cpe-*negative strains was tested using the Wilcoxon Rank Sum test (Rey and Neuhäuser, 2011).

## Results

### Distribution of Genes Encoding Virulence Factors Among *C. perfringens* Food Isolates

We successfully applied real-time PCR targeting 17 genes encoding 15 toxins or virulence factors on all 141 *C. perfringens* strains from 42 FBOs in order to discriminate virulence gene profiles. The distribution of genes encoding virulence factors shown in Table 4 was diverse among *C. perfringens* food strains. Four genes encoding virulence factors including *cpa*, *colA, nanH* and *cadA* were conserved in all tested isolates and were not included in Table 4. Similarly, the *cpb, etx, netB* and *tpeL* genes were not detected in any of the analyzed strains and were therefore excluded from Table 4. According to the new toxin-based classification system (Rood et al., 2018), toxinotype F was the most prevalent and represented 79 strains (56%). Sixty-one strains were classified as toxinotype A and only one strain belonged to toxinotype E. However, our analysis provided a much more contrasted view of the foodborne strains. In total, 15 virulence gene profiles from I to XV were obtained. The strains harbored 4 (profile XV) to 11 (profile I) virulence genes. Six profiles of virulence genes (I, III, IX, XII, XIV, and XV) were represented by only one strain. Interestingly, profile I was represented by a strain belonging to *C. perfringens* type E (i.e., presence of the *ia* and *ib* genes encoding iota toxin).

**Table 4:** Profiles of virulence genes identified in *C. perfringens* strains isolated in the Paris region from 2013 to 2017 (*n* = 141).

Virulence gene profile	Number of strains	Genes detected									Toxinotype
			
		*cpb2*	*ia*	*Ib*	*cpe*	*pfoA*	*lam*	*nagH*	*nanI*	*nanJ*	
I	1	-	+	+	+	+	-	+	+	+	E
II	9	+	-	-	+	+	-	+	+	+	F
III	1	-	-	-	+	+	-	+	+	+	F
IV	12	-	-	-	+	-	-	+	-	+	F
V	24	-	-	-	+	-	-	+	-	-	F
VI	4	-	-	-	+	-	-	-	-	+	F
VII	29	-	-	-	+	-	-	-	-	-	F
VIII	7	+	-	-	-	+	-	+	+	+	A
IX	1	+	-	-	-	+	-	-	+	-	A
X	40	-	-	-	-	+	-	+	+	+	A
XI	5	-	-	-	-	-	-	+	+	+	A
XII	1	-	-	-	-	+	-	-	+	-	A
XIII	5	-	-	-	-	-	-	+	-	-	A
XIV	1	+	-	-	-	+	+	+	+	+	A
XV	1	-	-	-	-	-	-	-	-	-	A


*The column toxinotype refers to the new toxin-based typing scheme (Rood et al., 2018). +, presence of gene; -, absence of gene.*

CPE is considered by many authors as the sole toxin responsible for the symptoms occurring during foodborne illness caused by *C. perfringens* (Brynestad and Granum, 2002; Lindström et al., 2011; Chon et al., 2012; Freedman et al., 2016). Consequently, we paid particular attention to the distribution of the *cpe* gene in our collection of strains (Table 4). In total, 80 strains were positive for the *cpe* gene and 61 were *cpe-*negative. The distributions of virulence genes were different according to the presence or absence of the *cpe* gene. Four profiles (IX, XI, XII, and XIV) with a combination of genes encoding virulence factors were detected only in *cpe*-negative strains. Conversely, three profiles (I, IV, and VI), which contain a combination of genes encoding virulence factors, were only detected in *cpe-*positive strains. Four profiles (II/VIII, III/X, V/XIII and VII/XV) were identical between *cpe*-negative and *cpe-*positive strains.

A significant trend emerged from the comparative analysis of *cpe*-positive vs. *cpe-*negative strains: 80% (49/61) of *cpe-*negative strains carried 8 to 10 virulence genes, while 86% (69/80) of *cpe*-positive strains carried only five to seven virulence genes (*p* < 0.05). The biological relevance of this observation can be assessed by performing *in vitro* cytotoxicity tests. Profile VII was the most common among *cpe*-positive strains (36%) and lacks all the other genes encoding virulence factors. Importantly, *C*. *perfringens* was the only pathogen identified in five of seven outbreaks where profile VII was detected, confirming the prominent role of CPE in pathogenicity. On the other hand, virulence gene profile X was the most prevalent among *cpe-*negative *C. perfringens* strains (66%, 40/61). Profile X carried the *pfoA*, *nagH, nanI*, and *nanJ* genes. The gene encoding perfringolysin O (*pfoA*), a well characterized pore-forming toxin, was found in 82% (50/61) of *cpe-*negative *C. perfringens* strains and was associated with hyaluronidases genes (*nagH)* in 48 of the 50 *cpe-*negative *pfoA-*positive strains. The Sialidase genes (*nanI* and *nanJ*) were also found predominantly in *cpe-*negative strains (87%, 53/61). An interesting finding is shown in Table 4: *nagH* is the only virulence gene present in profile XIII, which was the only strain isolated in large FBOs (outbreaks 2207 and 2253), suggesting that NagH could be a marker of the enterotoxigenic potential of *cpe*-negative strains. This hypothesis is compatible with the epidemiologic characteristics of *nagH cpe*-negative strains (profiles IX, XII, and XV), which were always found to be associated with *nagH*-positive profiles in the investigated outbreaks. However, this result could also be due to a fastest growth rate in food that could make *cpe+* strains undetectable. This hypothesis could be tested experimentally in competition growth assays. In addition and to clarify the potential pathogenicity of *cpe-* strains it would be interesting to perform PCR targeting the *cpe* gene on DNA extracted directly from the food mother suspension. The development of such a PCR test made on food suspension would complement the standard microbiological detection method.

### Profiles of Virulence Genes per Foodborne Outbreak

To explore the genetic diversity within the strains isolated in the same outbreak, we considered the 29 FBOs for which at least two to eight *C. perfringens* isolates per outbreak were recovered. A FBO is considered to be heterogeneous when two or more recovered isolates show different virulence gene profiles. Several profiles of virulence genes (i.e., up to three different profiles) were retrieved from 10 outbreaks. In addition, the *cpe* gene was not detected in seven of the 10 heterogeneous FBOs (outbreaks 2540, 2606, NK, 3803, 4115, 4493, and 529) (Table 2). *C. perfringens* was the sole pathogen identified in two outbreaks.

The isolated strains had homogeneous virulence gene profiles in 19 FBOs. The *cpe* gene was found to be present in 14 of the 19 homogeneous FBOs. In addition, *C. perfringens* was the only pathogen isolated in all homogeneous FBOs in which the *cpe* gene was detected. Among the homogeneous FBOs with *cpe-*positive strain profiles, profiles V and VII were the most commonly detected in nine out of 14 FBOs. Conversely, the *cpe* gene was not detected in 5 FBOs with homogeneous profiles of virulence genes.

Finally, only *cpe*-negative *C. perfringens* isolates were detected in 12 out of 42 FBOs using the horizontal method EN NF ISO 7937 for the enumeration of *C. perfringens* (Anonymous, 2005). This observation does not prove the pathogenicity of these strains since other pathogens may have escaped detection. However, we cannot exclude the possibility that *cpe*-negative *C. perfringens* isolates may have played a role in the diseases.

### Phylogenomic Inference

**FIGURE 1:**
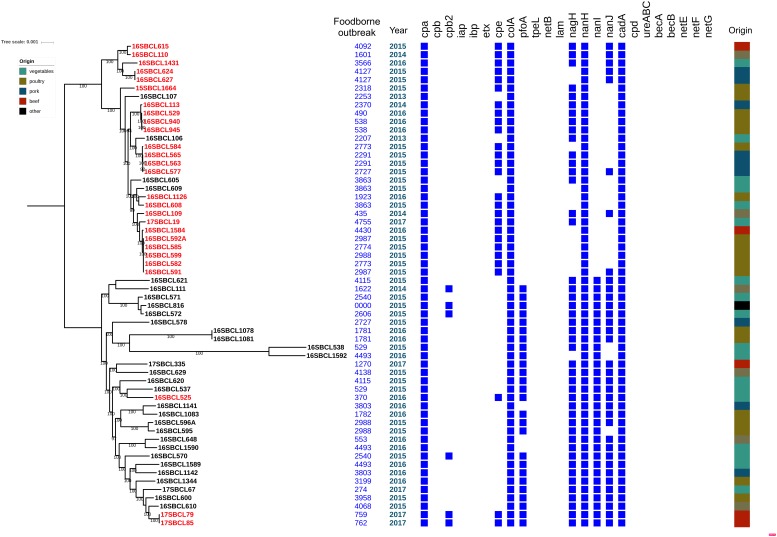
Coregenome phylogenomic inference of 58 *C. perfringens* isolates. The maximum likelihood tree was constructed using coregenome SNPs identified with iVARCall2. The phylogenomic history was inferred using RAxML and branches were supported by bootstrap analysis (*n* = 100). The phylogenetic inference converged after 50 bootstrap replicates. Metadata were visualized using iTOL. The presence of virulence genes is indicated with blue squares. The *cpe*-positive strains are in red. The large multi-colored strip indicates different food matrices (origin).

**FIGURE 2:**
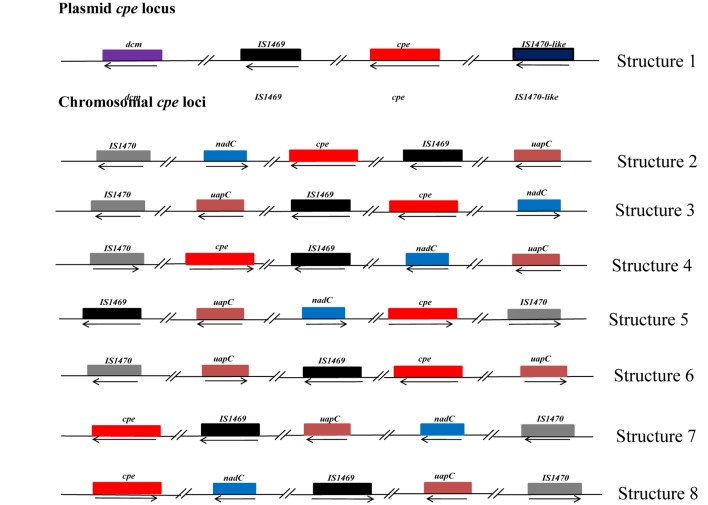
Description of the genetic organization of the *cpe* locus in *C. perfringens* strains. Each box represents an open reading frame. The plasmid-*cpe* locus (structure 1) includes the *dcm* gene and two characteristic insertions sequences, *IS1469* and *IS1470-like*. The sequence analyses indicated 99% identity with 100% query coverage with plasmid pCPF4969 of *C. perfringens* strain F4969. Seven other different structures of the *cpe* locus are shown. Analysis of these structures revealed that these loci are located in 3 Mbp, corresponding to the chromosomal *cpe* locus. However, the identified structures in our study are different with respect to dual IS1470 flanking the *cpe* gene, as described by Miyamoto et al. (2002). Structure 2 corresponds to 16SBCL584, 16SBCL582, 16SBCL577, 16SBCL592A, 16SBCL592B, 16SBCL1584, 16SBCL1126, 16SBCL529, 16SBCL591, 16SBCL585, 16SBCL1664, 16SBCL945, and 16SBCL599. Structure 3 corresponds to 16SBCL565, 16SBCL563, 16SBCL110, and 16SBCL615, structure 4: 16SBCL627 and 16SBCL1431, structure 5: 16SBCL113, structure 6: 16SBCL109 and 16SBCL624, structure 7: 16SBCL940 and structure 8: 17SBCL19.

Our PCR-based analysis focusing on the virulence gene profiles shows that our collection of FBO-associated *C. perfringens* strains is segmented into two genetically distinct clusters, namely *cpe*-positive and *cpe*-negative strains. In order to check the results obtained by PCR, we performed a whole genome analysis of strains representative of the diversity of the dataset. The study of genetic relationships was based on coregenome SNPs and the maximum-likelihood method to provide a strong phylogenetic analysis. The tree was robust with most branches of the backbone presenting high bootstraps values. Strikingly, two distinct clades were identified that distinguish most *cpe*-positive strains (24/27) from *cpe*-negative strains (27/30) (Figure 1). A closer examination of the genomic environment of the *cpe* locus in the 3 *cpe*-positive strains (16SBCL585, 17SBCL79, and 17SBCL85), which clustered in the *cpe-*negative clade, revealed that it is flanked by a putative cytosine methyltransferase gene (*dcm*) and two insertion sequences IS1469 and IS1470-like found in a 70 kb scaffold (Figure 2). By BLAST querying against plasmid NCBI database, we found 99.9% identity with plasmid pCPF4969 (Adams et al., 2015, 2018). This result indicates that the *cpe* gene is carried on a the pCPF4969 plasmid (Li et al., 2013).

A similar analysis performed among 24 *cpe-*positive strains identified seven different *cpe* loci which were all found in the 3.0 Mb scaffold corresponding to the chromosome. The chromosomal organizations (Figure 2) of the *cpe* loci correspond to a variety of arrangements of the IS1469, IS1470, *nadC*, *uapC*, and *cpe* loci (Figure 2). To our knowledge, shuffling at the *cpe* locus has not previously been described and could have consequences on CPE production.

### Genomic Analysis

**FIGURE 3:**
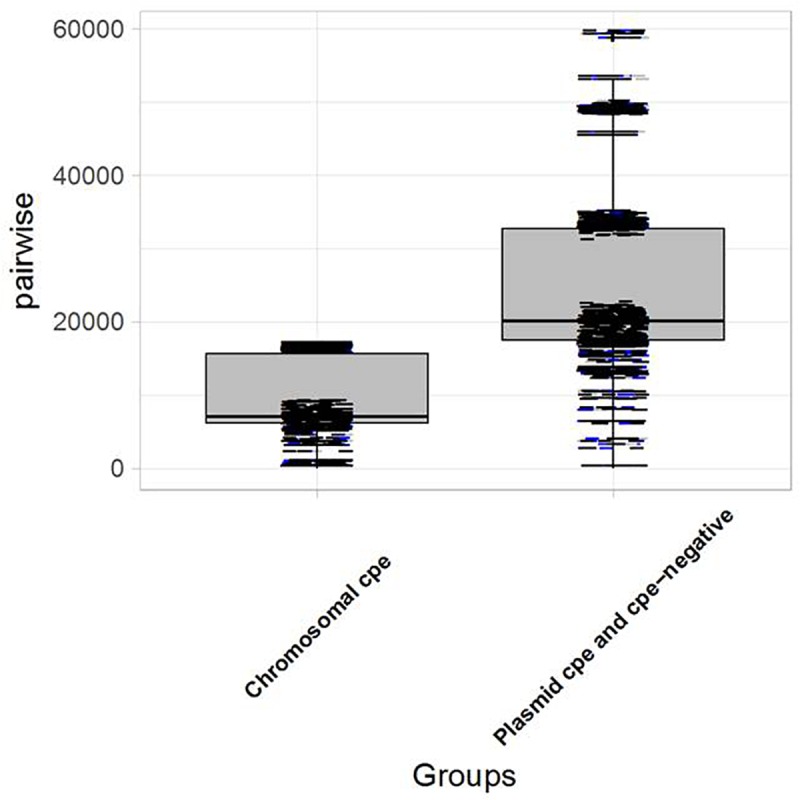
Boxplot showing the distribution of strains carrying the *cpe* gene on the chromosome vs. plasmid-*cpe* and *cpe-*negative strains. The Kolmogorov–Smirnov test yields *D* = 0.83266, and *p*-value < 2.2e-16, indicating a significant difference in terms of genetic diversity between the pairwise SNP differences of the two groups.

Genomic analysis revealed unexpected links between different outbreaks. The pairwise SNP differences were used to establish the level of relatedness among the strains. A comparative analysis of intra-clade genetic diversity was performed. An average pairwise SNP difference of 8,494 ± 499 was calculated within chromosomal *cpe-*positive strains, compared to 21,718 ± 901 within *cpe-*negative and plasmid-*cpe* strains. The genetic diversities of these two clusters were significantly different in terms of pairwise SNP differences (*p* < 0.05, Figure 3).

We compared the genetic relatedness of strains isolated from the same FBOs and presenting the same profile of virulence genes. We found that the strains were indistinguishable in three out of four cases, indicating homogeneous contamination. On the other hand, two co-contaminated strains were detected with a highly unrelated pairwise distance of 6,484 SNPs (FBO2773). This result indicates that contamination with a heterogeneous profile can occur and that virulence gene profiling is not a sufficiently discriminatory criterion, in particular to differentiate strains during FBO investigations.

Nucleotide BLAST was used to identity and confirm virulence factors previously detected by real-time PCR, including the *becA*, *becB*, *cpd*, *ureABC, netE*, *netF* and *netG* genes. For the 58 sequenced *C. perfringens* isolates, the results obtained by BLAST were similar to those obtained by real-time PCR detection. These results confirm the specificity and robustness of our PCR systems. Our *in silico* analyses were negative for *becA* and *becB* encoding BEC toxin, *ureABC* encoding ureases, *cpd* encoding lambda, and the recently identified pore-forming toxin genes *netE, netF* and *netG* in the 58 genomes.

## Discussion

*C. perfringens* has been recorded as the fourth most common cause of FBOs in France (Santé Publique France, 2016). FBO caused by *C. perfringens* results from the ingestion of foods that are contaminated with spore or vegetative cells (Brynestad et al., 1997). The true human burden of *C. perfringens* is likely much higher as many outbreaks are never investigated and many illnesses occur sporadically (Vaillant et al., 2005). Our study shows that FBOs were commonly due to meat and meat product foods (52%, 22/42), which is consistent with the epidemiology of *C. perfringens* (Wen and McClane, 2004; Grass et al., 2013). The contamination of meats may occur through contact of carcasses with feces, as well as via cross-contamination by other foods or contaminated surfaces during slaughtering. Although most *C. perfringens* FBOs are attributed to meat and poultry products (Grass et al., 2013), our study shows that in eight cases *C. perfringens* was the only pathogen isolated from vegetable dishes. This unexpected finding is likely linked to the ubiquitous nature of *C. perfringens*, which can be isolated in the environment and the soil (Uzal et al., 2014). Improper holding temperatures and incomplete cooking of foods are recognized as major factors contributing to the development of *C. perfringens* FBOs (Sarker et al., 2000a). Moreover, vegetables may initially be contaminated by spores of *C. perfringens* (Xiao et al., 2012), while the cooking temperature of vegetable dishes is too low to destroy spores (Talukdar et al., 2016). *C. perfringens* can grow at temperatures from 20 to 53°C and spores can survive high temperatures (up to 95°C for 1 h) (McClane et al., 2013). We observed that certain virulence gene profiles (V, VII, X, see Table 4) are prevalent in our collection. It would be of interest to measure the heat resistance of a selection of isolates to determine if this phenotypic trait can explain the prevalence of specific genomic profiles in food.

Symptoms due to foodborne *C. perfringens* are similar to those caused by other foodborne pathogens, especially *B. cereus* diarrheal strains (Tewari and Abdullah, 2015). Moreover, current methods for investigation of *C. perfringens* in FBOs are based on enumeration of characteristic colonies in specific media, followed by biochemical confirmation. These methods are useful to collect strains but do not provide any typing information. As mentioned above, the revised typing scheme classifies strains producing CPE as type F. However, understanding the role and virulence of *C. perfringens* strains isolated in the context of FBOs requires us to describe the genetic diversity of the implicated strains and to develop robust and novel typing methods. In the present work, we developed a new PCR-based technique on a comprehensive set of 17 genes encoding putative virulence and toxin factors. This new PCR-based technique was used to characterize the diversity of a collection of 141 strains implicated in 42 FBOs.

The *cpa*, *colA*, *nanH*, and *cadA* genes encoding α-toxin, κ-toxin, sialidase (i.e., a small intracellular sialidase with 43 kDa of molecular weight) and deoxyribonuclease were found to be conserved in all isolates analyzed. Previous genomic studies support this result (Goossens et al., 2017; Kim et al., 2017; Kiu et al., 2017). Consequently, these genes cannot be considered relevant markers to discriminate FBO isolates. Conversely, four genes were not identified in our collection. Consequently, our typing scheme was reduced to nine genes and was used to characterize the virulence profile of a collection of 141 *C. perfringens* strains implicated in 42 FBOs. Fifteen different profiles were recognized (Table 4), demonstrating the discriminatory power of this method. The epidemiological relationships between *C. perfringens* of different origins, including isolates from FBOs, have been investigated previously (Jost et al., 2006; Afshari et al., 2016). The previously published articles focusing on FBO isolates and animals concluded that isolates from the same outbreak have a similar pattern, while genetic diversity is high in non-outbreak isolates selected randomly (Johansson et al., 2006; Chalmers et al., 2008b; Fohler et al., 2016). Our results challenge this view and demonstrate that a range of virulence gene profiles can be found within the same FBO.

A more thorough examination of *C. perfringens* diversity revealed interesting patterns. Except for profile I, the presence of *cpe* (*p* < 0.05) was associated with a low number (5–7) of other virulence genes. More precisely, a strong association was observed between the presence of *cpe* and the lack of *pfoA* and *nanI* (*r* = -0.67, *p* < 2.2 × 10^-16^). This observation is consistent with previous studies showing that most *cpe*-positive *C. perfringens* strains lack the *pfoA* gene (Deguchi et al., 2009). The presence of the *pfoA* and *nanI* genes was found to be significantly associated (61/66; *p* < 2.2 × 10^-16^). The most predominant profiles in *cpe*-negative strains were the profiles X, with 40 of the 61 strains, and VIII, with seven of the 61 strains. In these two profiles, the *pfoA* gene was associated with sialidases and/or hyaluronidase. These toxins could contribute to the overall virulence of the bacterium (Revitt-Mills et al., 2015). Previous *in vitro* studies have demonstrated a synergic effect of perfringolysin O and α-toxin on the cytotoxicity of non-foodborne strains (Awad et al., 2001); the association between *pfoA* and *nanI* could have toxicologic consequences that would be worth studying in an appropriate experimental system.

Profiles IV, V, and VII were predominant and were found in 18 of the 29 FBOs with *cpe*-positive profiles. In addition, profile VII carries *cpe* as the sole toxin-encoding gene. These results confirm that CPE is a strong contributor to foodborne illness due to *C. perfringens* (Sarker et al., 2000b).

Two to three different profiles of virulence genes were identified in three FBOs where *C. perfringens* was the only isolated pathogen, demonstrating that *Clostridium* FBOs can be associated with heterogeneous contamination of food. This important result complicates the epidemiology of clostridial FBOs, and the contribution of the different gene profiles to the development of symptoms needs to be investigated further. A direct consequence is that pathogenic strains co-infecting foods with non-pathogenic strains can be overlooked. It is therefore important to couple a PCR-based method targeting genes with the NF EN ISO 7937 standard method and characterize more than five strains isolated in the same FBO.

To further analyze the diversity of *C. perfringens-*associated FBOs, WGS of 58 isolates of *C. perfringens* was conducted to provide insight into the phylogenomic relationships and distribution of virulence factors among *cpe-*positive and *cpe-*negative strains. To the best of our knowledge, this study represents the first large genomic analysis focusing on *C. perfringens* food isolates, compared to other studies screening other sources and including few foodborne samples (Hassan et al., 2015; Kiu et al., 2017). The computed coregenome phylogenomic inference indicates that *C. perfringens* strains carrying the *cpe* gene on the chromosome have evolved independently from *cpe*-negative and plasmid carrying *cpe* strains. This argues for the possibility of its involvement in adaptation to specific niches, and supports previous research conducted on *C. perfringens* strains from different origins showing a clear partitioning of strains capable of inducing foodborne illness into lineages distinct from those of strains isolated from other environments (Hibberd et al., 2011). Previous studies (Miyamoto et al., 2002; Li et al., 2013) have localized the *cpe* gene near the *dcm* gene on both the pCPF4969 and pCPF5603 plasmids of type A isolates. Our investigation of the genetic environment of 16SBCL525, 17SBCL79, and 17SBCL85 is consistent with these previous studies and demonstrates that the *dcm* gene is proximal to the plasmid-borne *cpe* gene. The fact that these strains were phylogenetically clustered with *cpe-*negative strains supports the notion that the *dcm* region of plasmids represents a hot-spot for insertion of certain mobile genetic elements, including some carrying a *cpe* gene. An early study indicated that *C. perfringens* carrying the *cpe* gene on the chromosome is flanked by upstream and downstream *IS1470* and, instead of a *dcm* gene, they carry the purine permease (*uapC*) and quinolinate phosphoribosyltransferase (*nadC*) genes on the chromosome (Brynestad et al., 1997). Our investigations support this assertion and, to our knowledge, this is the first study describing a diversity of genetic arrangements of the *cpe* locus in chromosomal *cpe* strains (Brynestad et al., 1997; Miyamoto et al., 2002; Li et al., 2010).

The coregenome phylogenomic reconstructions provide interesting epidemiologic information and indicate actual relationships between strains from different outbreaks. FBOs 2987, 2774, 2988, and 2773 were caused by closely related strains with regard to a limited average of pairwise distances between them (i.e., 7.0 SNPs) and common profiles of virulence genes. This similarity could be due to the same biotype that may reach food and cause FBOs. Due to wide biotype and ecological niches, genetically diverse strains of *C. perfringens* may be found in food matrices. Important epidemiologic findings have resulted from the high resolution of genome sequencing. For instance, WGS was used to demonstrate that many *C. difficile* cases could not be attributed to transmission from asymptomatic patients, but that there is high diversity of *C. difficile* in the environment (Tang et al., 2017). This approach could be used in our analysis. We have identified considerable genetic diversity within *cpe-*negative strains. The high genetic diversity may in part be explained by the broad distribution of this group in the environment. An earlier genomic comparison study conducted on three genomes of *C. perfringens* (ATCC13124, SM101, and strain 13) revealed considerable genomic diversity with more than 300 unique genomic islands identified (Myers et al., 2006). Our phylogenomic analysis supports this notion with regard to strains 16SBCL582 and 16SBCL584 from FBO 2273. However, a comparison of the virulence gene profiles of these distant strains (obtained by real-time PCR) indicates that they present the same virulence gene profile. This result suggests that the approaches based on the detection of virulence factors are not sufficient to efficiently discriminate *C. perfringens* strains.

Another interesting finding is that strains are grouped according to the distribution of virulence factors. In particular, the presence of the *nanI*, *pfoA* and *nagH* genes in *cpe-*negative strains is of interest, and it would be worthwhile to investigate the putative role of these virulence factors in *cpe-*negative disease.

## Conclusion

The aim of our study was to characterize *C. perfringens* involved in FBOs from the Paris region between 2013 and 2017 by investigating profiles of virulence genes according to the presence or absence of 17 genes encoding 15 virulence factors screened by a new real-time PCR tool. High diversity was obtained between strains in different outbreaks. Heterogeneous profiles of virulence genes within the same outbreaks were highlighted for the first time, indicating that food may be contaminated with different *C. perfringens* strains that may or may not harbor the *cpe* gene. Our study emphasizes the importance of more detailed characterization of *C. perfringens* isolates involved in FBOs than simple enumeration on specific agar, as suggested by the EN ISO 7937 reference method and/or characterization of five random isolates.

We have shown that the reconstructed coregenome phylogenomic history of *C. perfringens* food isolates can identify two mains groups, both being able to contaminate food. Our research did not clearly associate food sources with a specific clade. However, future comparative genomic studies on strains of various origins and from different geographical areas should provide clues to understand *C. perfringens* pathogenicity and associations to ecological niches. Genomic studies combined with epidemiological data provide new leads to design toxicity assays that may renew our understanding of the molecular basis of C. *perfringens* pathogenicity, and ultimately identify new biomarkers to differentiate between pathogenic and non-pathogenic *C. perfringens* strains. Finally, it would be interesting to characterize *C. perfringens* strains implicated in the FBO with respect to their reservoir.

## Author Contributions

AMA conceived the study, designed the experiments, and wrote the manuscript. NR approved bioinformatics analyses, provided advice for statistical analyses, and revised the manuscript. AMA and KH performed all bioinformatics analyses. MLN contributed to the microbiologic analyses. SaD, PF, and SoD conceived the methodology and performed high throughput real-time PCR analyses. OF, J-AH, and M-YM conceived and piloted the project and revised and approved the manuscript for publication.

## Conflict of Interest Statement

The authors declare that the research was conducted in the absence of any commercial or financial relationships that could be construed as a potential conflict of interest.

## Footnotes

1 www.icm-institute.org
