# Erratum: Large-Scale Genomic Analyses and Toxinotyping of *Clostridium perfringens* Implicated in Foodborne Outbreaks in France

**DOI:** 10.3389/fmicb.2019.01564

**Published:** 2019-07-03

**Authors:** Frontiers Production Office

**Affiliations:** Frontiers Media SA, Lausanne, Switzerland

**Keywords:** foodborne outbreak, *Clostridium perfringens* enterotoxin, virulence gene profiles, virulence factors, real-time PCR, whole genome sequencing, coregenome SNP

Due to an editorial error, the Data Availability statement containing the accession numbers of the sequenced strains was erroneously omitted from the article.

The publisher apologizes for the mistake.

## Data Availability

Read sequences used in this study were submitted online on the NCBI network (https://www.ncbi.nlm.nih.gov/biosample) under the accession numbers: SAMN09721416, SAMN09721417, SAMN09721418, SAMN09721419, SAMN09721420, SAMN09721421, SAMN09721422, SAMN09721423, SAMN09721424, SAMN09721425, SAMN09721426, SAMN09721427, SAMN09721428, SAMN09721429, SAMN09721430, SAMN09721431, SAMN09721432, SAMN09721433, SAMN09721434, SAMN09721435, SAMN09721436, SAMN09721437, SAMN09721438, SAMN09721439, SAMN09721440, SAMN09721441, SAMN09721442, SAMN09721443, SAMN09721444, SAMN09721445, SAMN09721446, SAMN09721447, SAMN09721448, SAMN09721449, SAMN09721450, SAMN09721451, SAMN09721452, SAMN09721453, SAMN09721454, SAMN09721455, SAMN09721456, SAMN09721457, SAMN09721458, SAMN09721459, SAMN09721460, SAMN09721461, SAMN09721462, SAMN09721463, SAMN09721464, SAMN09721465, SAMN09721466, SAMN09721467, SAMN09721468, SAMN09721469, SAMN09721470, SAMN09721471, SAMN09721472, SAMN09721473.

