# Supplementary material for: Large-Scale Genomic Analyses and Toxinotyping of Clostridium perfringens Implicated in Foodborne Outbreaks in France
Source: Front Microbiol. 2019 Apr 17;10:777. doi: 10.3389/fmicb.2019.00777 (PMC6481350; doi:10.3389/fmicb.2019.00777)
Supplement: Supplementary file 1 [file Table_1.docx]

Supplementary Material

**Large-scale Genomic Analyses and Toxinotyping of *Clostridium perfringens* Implicated in Foodborne Outbreaks in France**

Abakabir Mahamat Abdelrahim^*^, Nicolas Radomski, Sabine Delannoy, Sofia Djellal^1^, Marylène Le Négrate Katia Hadjab, Patrick Fach, Jacques-Antoine Hennekinne, Michel-Yves Mistou^1^ and Olivier Firmesse^*^

Université PARIS-EST, Agence Nationale de Sécurité Sanitaire, de l’Alimentation, de l’Environnement et du Travail (ANSES), Laboratory for Food Safety, Maisons-Alfort, France

Olivier.FIRMESSE@anses.fr

# Supplementary Table 1

List of complete closed reference genomes of *Clostridium perfringens.*

| **List of reference genomes** | | | | | |
| --- | --- | --- | --- | --- | --- |
| **Organism/strain** | **BioSample** | **Level** | **Size (Mb)** | **GC %** | **Genes** |
| *C. perfringens* ATCC13124 | SAMN02604008 | complete | 3.25668 | 28.40 | 2948 |
| *C. perfringens* Forc_003 | SAMN03140316 | complete | 3.39511 | 28.38 | 3077 |
| *C. perfringens* Str 13 | SAMD00061119 | complete | 3.08574 | 28.55 | 2791 |
| *C. perfringens* JP55 | SAMN03372134 | complete | 3.57123 | 28.21 | 3287 |
| *C. perfringens* Forc_025 | SAMN04209542 | complete | 3.34382 | 28.50 | 3055 |
| *C. perfringens* SM101 | SAMN02604026 | complete | 2.96009 | 28.18 | 2753 |

# Supplementary Figures 1


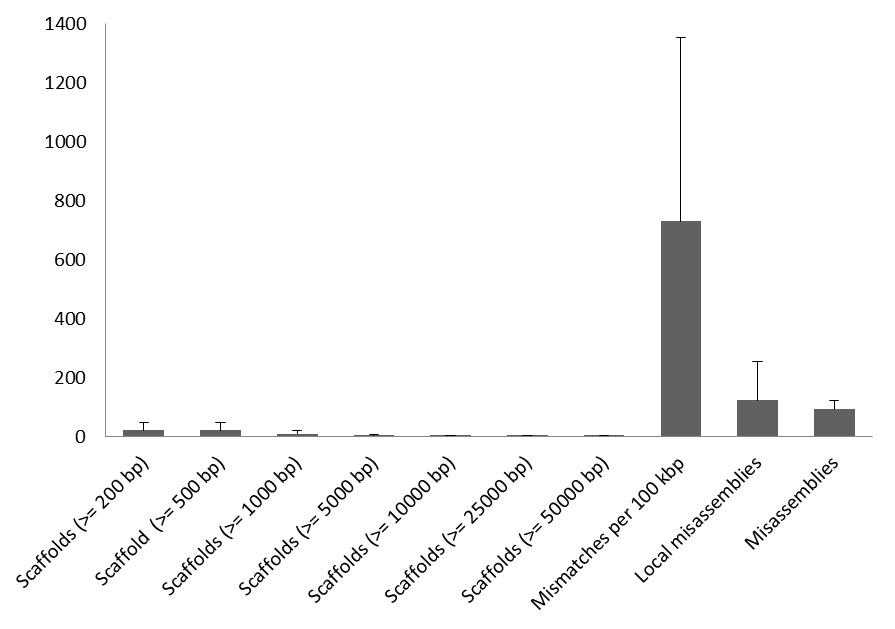

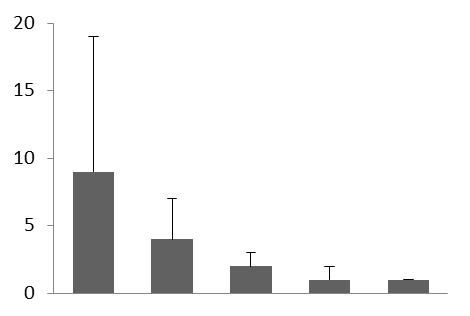

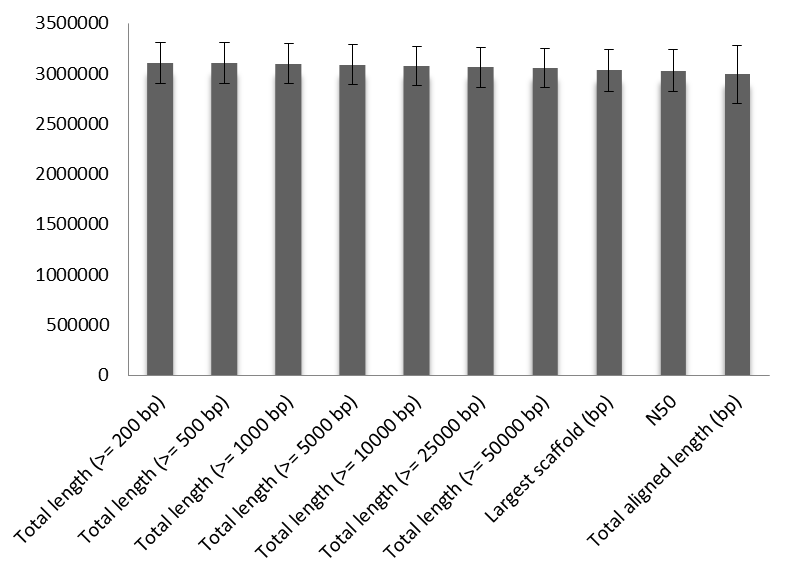


A

B

Parameters for quality assessment of 58 *C. perfringens* assembled genomes. (**A**) shows the number of contigs and (**B**) the size of scaffolds.
